# Post orgasmic illness syndrome successfully managed with antihistamine: A case report

**DOI:** 10.1016/j.eucr.2022.102189

**Published:** 2022-08-18

**Authors:** Andrew Shanholtzer, Jacob R. Stephens, Carl Lauter, Kenneth M. Peters

**Affiliations:** a Oakland University William Beaumont School of Medicine, United States; b Department of Urology, William Beaumont Hospital, Royal Oak, MI, United States; c Department of Allergy and Immunology, William Beaumont Hospital, Royal Oak, MI, United States

**Keywords:** Orgasm disorder, Sexual dysfunction, Post orgasmic illness syndrome

## Abstract

Post orgasmic illness syndrome (POIS) is increasingly being recognized as a debilitating cause of sexual dysfunction in males. It is often misdiagnosed due to its unfamiliarity to providers, resulting in numerous potentially unnecessary tests and treatments. Currently, there is no known single most effective treatment, but several case reports suggest desensitization, hormonal therapy, and other treatment modalities may be useful. However, these treatments are experimental in nature and have not been evaluated in placebo-controlled trials. We report on the use of a simple over-the-counter antihistamine in the management of POIS.

## 1 Introduction

Post-orgasmic illness syndrome, or POIS, is a very rare condition that was first described within the last twenty years and has less than sixty reported cases in the literature. The presentation is variable but can be characterized by the following common features: flu-like or allergic-like symptoms immediately following ejaculation which can last for up to seven days before spontaneous resolution.^1^ It has perhaps been best studied by Waldinger et al.^1^, ^2^, ^3^ who reported their experience in forty-five Dutch men.^1^ He and his associates proposed the following five criteria present in nearly all patients suffering from POIS:

1) One or more of the following symptoms: sensation of a flu-like state, extreme fatigue or exhaustion, weakness of musculature, experiences of feverishness or perspiration, mood disturbances and/or irritability, memory difficulties, concentration problems, incoherent speech, congestion of nose or watery nose, itching eyes.
2) All symptoms occur immediately (e.g. second), soon (e.g. minutes), or within a few hours after ejaculation that is initiated by coitus, and/or masturbation, and/or spontaneously (e.g. during sleep).
3) Symptoms occur always or nearly always, e.g. in more than 90% of ejaculation events.
4) Most symptoms last for about 2–7 days.
5) The symptoms disappear spontaneously.

We report our experience with a patient presumed to be experiencing POIS who was successfully treated with an over-the-counter antihistamine.

## 2 Case presentation

Our patient is a 27-year-old previously healthy male who presented with a chief complaint of flu-like symptoms occurring with ejaculation. He developed coughing, rhinorrhea, sneezing, as well as a hive-like rash on his forearms with ejaculation. Additionally, he noticed facial and cervical lymphadenopathy which worsened with increased frequency of ejaculation. He believes his symptoms began around age 18 after a case of suspected acute epididymitis which was treated empirically with trimethoprim-sulfamethoxazole. His symptoms occurred regardless if ejaculation was from masturbation or from sexual intercourse. He had a chlamydial infection two years prior to presentation that was treated appropriately and confirmed to have resolved. Because of the distressing nature of his symptoms, he actively avoided any sexual activity or romantic relationships.

Prior to presentation, he was evaluated by several providers including three urologists, an otolaryngologist, an infectious disease specialist, and an allergist. During his first encounter with an allergist, he was diagnosed with hay fever. He underwent allergen testing demonstrating a cantaloupe allergy and sensitivity to poison ivy. Immunoglobulin testing revealed an elevated IgE level of 257 IU/mL (normal: <99 IU/mL).

Aside from abstaining from sexual activity, the patient was treated with antibiotics without any symptomatic improvement. He had several unremarkable scrotal ultrasounds, a normal semen analysis, and a testosterone level of 421 ng/dL (normal: 249–836).

During our initial encounter, we recommended a trial of daily over-the-counter diphenhydramine while gradually increasing the frequency of ejaculation. Additionally, we referred him to an allergist specializing in immunology for possible autologous skin prick testing which has not been performed as of this report due to the COVID-19 pandemic. He eventually was prescribed fexofenadine 180 mg daily which led to a patient-reported 90% decrease in his post-ejaculatory symptoms, including the rash and lymphadenopathy, and this has allowed him to resume sexual activity.

## 3 Discussion

POIS is becoming increasingly recognized as a culprit among men experiencing sexual dysfunction. However, it remains a diagnosis unfamiliar to most providers and as such can present a diagnostic dilemma leading to an abundance of patient visits, laboratory testing, and imaging studies.

The exact etiology of POIS is unclear. Currently the best accepted theory is that symptoms result from Type I and Type IV allergic reactions to autologous semen.^1^ This theory is supported by both the clinical manifestations of POIS as well as the fact that 88% of men suspected to have POIS had positive skin-prick tests to diluted, autologous semen.^1^ While sperm are potentially immunogenic given the blood-testis barrier and genetic differences compared to one's own DNA, POIS has affected even those men who have undergone sterilization^1^ suggesting that if an immune response is the cause of POIS, the antigen is likely within the seminal fluid and not the semen itself. In our patient, whose symptoms seemed to develop after suspected epididymitis, it is possible that the infectious process acted as an insult to the blood-testis barrier, allowing an autoimmune process to occur.

POIS is such a rarely diagnosed disease and there is no known single most effective therapy. There have been reports of various treatments from selective serotonin reuptake inhibitors to benzodiazepines to immunotherapy.^3^^,^^4^ Waldinger et al.^2^ attempted desensitization by injecting patients with diluted autologous semen, progressively increasing the concentration based on clinical response. They reported one patient experiencing 60% symptomatic improvement over 31 months with improvement in premature ejaculation, while another patient experienced 90% symptomatic improvement over 15 months. While an attractive treatment option, this treatment is costly, requires frequent office visits for injections, and many providers may not be experienced or comfortable with injecting semen. Treatment with immunotherapy was trialed in a Brazilian patient with POIS with minimal symptomatic improvement.^4^ One patient with POIS and low normal testosterone was successfully treated with hCG.^5^

In our patient, the treatment that proved most efficacious was daily fexofenadine, leading to a 90% symptom improvement. Interestingly, when our patient took diphenhydramine, he did not achieve much symptomatic improvement despite similar mechanisms of action. This could be explained by the short half-life of diphenhydramine and a short time to peak serum concentrations of 2 h. Thus, in order to maintain adequate levels, doses every 2–3 hours around the clock would be required which could result in significant sedation and impairment. In contrast, fexofenadine is long acting and non-sedating, making it easier tolerated on a regular regimen.

Fexofenadine is a relatively safe, inexpensive, and well tolerated medication, but it requires more study in POIS before its therapeutic benefits in this select population can be assessed. Our experience demonstrates the feasibility of treating a complex disease with a simple medication and hopefully will be replicated in future patients.

## Funding

This research did not receive any specific grant from funding agencies in the public, commercial, or not-for-profit sectors.

## Declaration of competing interest

AS, JRS and CL have no conflicts of interest or disclosures. KMP is a co-founder of Micron Medical and serves as a consultant for Urogen and Urovant.
